# What evidence exists on conceptual differences in climate change perceptions of smallholders? A systematic map protocol

**DOI:** 10.1186/s13750-022-00284-w

**Published:** 2022-09-16

**Authors:** Lia Taruiap Troncarelli, Carla Morsello

**Affiliations:** 1 Environmental Science Pos-Graduate Program, Energy and Environment Institute, University of São Paulo, Av. Prof. Luciano Gualberto, 1289, Cidade Universitária, São Paulo, CEP:05508-010 Brazil; 2 School of Arts, Sciences and Humanities, University of São Paulo, Rua Arlindo Béttio, 1000, São Paulo, CEP: 03828-000 Brazil

**Keywords:** Climate change awareness, Climate change communication, Climatic variability, Global warming, Indigenous people, Public perception, Smallholders, Small-scale societies, Risk perception

## Abstract

**Background:**

Climate change is affecting small-scale populations worldwide. Evidence of adverse effects has been reported for smallholders’ agriculture, hunting, fishing, and gathering products from natural ecosystems (non-timber forest products). To take precautions or deal with such problems (i.e. to adapt), smallholders need to perceive climatic changes. Acknowledging this need, the literature on this topic is vast. Despite that, authors adopt alternative concepts of climate change perception, which may hinder comparisons of results across studies. Hence, the review team aim to systematically map the literature usage of the climate change perception concept.

**Methods:**

This systematic map will follow the CEE guidelines and conform to the Reporting Standards for Systematic Evidence form. The review team will rely on five electronic databases of scientific publications—Scopus, Web of Science Core Collection, BASE—Bielefeld Academic Search Engine, Science Direct Elsevier and PubMed—with pre-tested search terms only in English. Publications will be filtered through the “articles only” and “English language” selections. Titles, abstracts, and full texts will then be screened using pre-defined eligibility criteria, including small-scale and indigenous populations inhabiting rural areas, as well as presenting explicitly or implicitly the concept of climate change perception. From articles meeting the eligibility criteria, the review team will extract and encode the data while selecting the full texts for reading. The review team will use a codebook pre-elaborated for encoding. No critical appraisal of study validity will be undertaken. Finally, a database with coded metadata of all studies in the map will be made available. The review team will present the evidence in a report map with text, figures, and tables, besides a catalogue of all identified perception definitions.

**Supplementary Information:**

The online version contains supplementary material available at 10.1186/s13750-022-00284-w.

## Background

Climate change already affects all world regions, as human activities have caused the warming of soils, oceans, and the atmosphere in the last two thousand years. Alongside temperature increases, since the 1950s, the probability of extreme events such as heatwaves, intense drought, heavy rainfalls, floods, and tropical cyclones has risen [1]. For the future, the Intergovernmental Panel on Climate Change (IPCC) predicts that global warming will inevitably exceed from 1.5 to 2 °C during the twenty-first century unless carbon dioxide and other greenhouse gas emissions are significantly reduced in the coming decades [1, 2]. Climatic changes pose risks to health, economic activities, food security, water supply and threaten the human livelihoods, particularly vulnerable populations living in developing countries [1]. Foremost, global warming is expected to menace rural people, especially indigenous peoples and other smallholders who directly depend upon natural resources for their livelihoods [3].

Climate change already negatively impacts the livelihoods of small-scale populations [4]. To illustrate, among small-scale agriculturalists, higher temperatures and lower rainfall have increased agricultural crop pests in Bolivia [5], and have changed planting schedule in Bangladesh [6]. In the Peruvian Amazon, more intense floods reduced wildlife populations and, consequently, wild meat consumed by indigenous people [7]. Among indigenous hunters, ice retreat in Alaska impaired cultural practices associated with subsistence, threatening food security [8]. For indigenous people in the Canadian Arctic, climate unpredictability and thinner unstable ice have reduced wild meat and fish consumption, lowering nutrient intake [9]. In the western Himalayas, changes in temperature have diminished the availability of non-timber forest products (NTFPs) among small-scale horticulturalists [10]. Although the current scenario is problematic, when smallholders perceive climatic changes, they may mitigate their negative impacts. Indeed, smallholders’ long-term connection and continuous interactions with natural environments, besides their traditional knowledge, may help them detect, understand, and face environmental changes [11].

Smallholders can take precautions or deal with climate change problems through adaptation strategies. Adaptation refers to people’s responses to expected or in progress impacts, which individuals implement, private companies, or governments to contain, avoid damage, or even take advantage of potential benefits [12]. Adopting such strategies necessarily begins with the affected population’s perception of ongoing climatic changes [13, 14]. Only by being aware of those affected can they assess whether they can respond to the observed changes by adopting any adaptation strategy if and when necessary [15]. Furthermore, investigating people’s perceptions is important. This contributes to identifying impacts or changes neglected in the scientific literature, a feature important in places where meteorological data are non-existent or inadequate [16].

In summary, investigating if, when, and how small-scale populations perceive climate change is essential, and scientists have responded accordingly with a profusion of studies on the topic. Despite that, several studies on this topic do not adopt an explicit or at least a clear definition of perception (e.g. [17–23]).

Formally, the definition of perception originates in Psychology, particularly in Cognitive Psychology [24, 25], which investigates mental processes. In this discipline, people’s perception is usually defined as the set processes by which an individual recognises, organises, and makes sense of the sensations he/she receives from environmental stimuli [25]. Although the term may have different meanings even within Psychology, a common element in most perception concepts is that it involves the analysis of sensory information. Thus, when cognitive psychologists talk about perceptions, they usually refer to the basic cognitive processes involved in analysing information received from people’s senses [26].

In contrast, in the rare studies on climate change perception that do provide a definition (e.g. [27–32]), conceptualisations of people’s perceptions are diverse and encompass not only sensory perceptions [6, 28, 31, 33, 34], but also people’s subjective interpretations [35], knowledge [32], awareness and comprehension of the environment [27, 36], beliefs about ongoing changes [29, 32], experiences towards climate variability [32], as well as concern and affect to it [37].

Due to this diversity of definitions about climate change perception, comparisons and syntheses of the scientific literature are hindered for at least three reasons. First, various thematic areas address the concept of climate change perception but are not restricted to it. For instance, the concept is incorporated in studies about traditional/local knowledge [20, 38] or its comparison with actual meteorological data [35, 39], adaptation strategies [14], cognitive biases [18], and perception drivers [40]. Second, studies diverge on the sample units adopted to investigate perceptions, ranging from individuals, households, and communities. Third, studies rely on different methods of investigation (qualitative, quantitative or mixed) [41], with implications for how the results can be interpreted and the concept of perception defined.

Thus, this article provides a systematic mapping protocol for synthesising information on the alternative definitions of climate change perception. Understanding how climate change perception has been conceptualised may contribute to standardising term usage across this subject literature. If so, information syntheses and comparisons would be more reliable, which are essential in this subject area to devise adaptation policies, particularly in smallholder societies’ contexts.

## The objective of the review

This systematic map aims to identify, classify, and describe the available evidence on the various underlying concepts adopted by the scientific literature to refer to climate change perceptions. The review team (hereafter, team) will focus on the literature addressing small-scale rural populations, including those of indigenous origin, because they directly depend on natural resources. Thus, these groups are more likely to suffer from the adverse effects of climate change [3] and benefit from climate change perceptions to adapt their subsistence practices. Results from this review may contribute to advance research and practice related to climate change. Although the scientific literature abounds with statements attesting to the importance of people’s perceptions to climate change adaptation, studies use the same term with various meanings. Therefore, it hinders the organisation and comparison of the current empirical literature to (i) evaluate which forms of perception are essential to adaptation, and (ii) to identify critical knowledge gaps where future evaluations and syntheses are needed. Improved knowledge about the role of people’s perceptions of climate change according to specific definitions may also contribute to better policies aimed at increasing small-scale populations’ adaptation.

The primary research question of this study is, therefore:

*“What evidence exists on the alternative definitions of climate change perceptions adopted in the literature about small-scale populations?”*

The secondary research questions are as follows:

*“How do the definitions of climate change perception vary and are interpreted across articles, according to their thematic areas, populations of interest, and geographical origin?*

*What constructs differentiate one definition from another? Are there similarities?”*

The components of the primary question are:

Population (P): Small populations living in rural areas, which consist of individuals who produce their resources for livelihood through family labour, with little or no ability to generate surplus production for the market [42].

Exposure (E): Climate change will be considered as exposure.

Outcome (O): Climate change perception definitions.

## Methods

This systematic mapping will: (i) follow the Collaboration for Environmental Evidence guidelines (CEE) [43] and (ii) conform to the Reporting Standards for Systematic Evidence (ROSES) form (Additional file 1) [44]. Systematic mapping is a transparent, rigorous, and objective review technique (i.e. follows a well-defined protocol), which helps to reduce biases inherent to traditional narrative reviews [44, 45]. The mapping technique consists of cataloguing, collating, and describing the existing literature on a particular yet broad topic [45, 46]. In our case, the team will identify and classify the various concepts adopted to refer to smallholders’ perceptions about climate change. The team will them describe how the literature conceptualises perceptions and whether this varies according to: (i) regional differences across the geographical space; (ii) subsistence strategy; (iii) description of phenomenon observed (physical phenomenon, biological phenomenon, human phenomenon). Unlike systematic reviews, assessment of evidence quality is optional in systematic mapping. As we will aimed to identify the various forms, correct or incorrect, adopted to refer to climate change perceptions, quality assessment was inappropriate. Hence, no quality assessment will be incorporated. The final database will be a metadata of studies describing bibliographic information, study area, origin and subsistence strategies of the investigated populations, thematic areas addressed in the articles, different definitions of climate change perceptions, constructs that are part of these definitions, the phenomena observed in these articles (physical, biological, human), information about sampling unit, data and methods.

Systematic mapping is appropriate for open-framed questions, such as ours. It is also more appropriate than systematic reviews when the studies to be collated are highly heterogeneous and were generated by different methodologies, such as a mixture of qualitative and quantitative research [45]. The evidence gathered from this approach may be used for developing conceptualisations on a specific topic, in our case, the perception of climate change.

### Searching for articles

The systematic mapping will rely on searches in five electronic scientific databases of scientific publications, namely “Scopus”, “Web of Science Core Collection” (WoS), “BASE—Bielefeld Academic Search Engine”, “Science Direct Elsevier” and “PubMed”. The team have chosen these databases because they are comprehensive, multidisciplinary, and peer-reviewed. They also encompass most of the publications on the Environmental Sciences domain that pertain to climate change perceptions. Additionally, the five selected databases implemented processes to guarantee publication quality. In WoS, there is a curation process by specialised and in-house expert editors who deal with a specific subject category. These editors have no affiliation with any publisher house or research institute to avoid biases. To be included in the WoS database, journals are evaluated with a set of 28 criteria [24 quality criteria and four impact criteria] [47]. Similarly, Scopus relies on an independent and international group of scientists and researchers with experience in journal editing to assess journal quality. These researchers are experts in their respective fields and, altogether, form the Scopus Content Selection and Advisory Board (CSAB) [48]. BASE provides over 240 million documents from over 8000 sources, with 60% of indexed documents open access. BASE indexes documents and journals that meet specific requirements for quality and academic relevance by qualified personnel from the Bielefeld University Library, in Germany [49]. Science Direct Elsevier database contains more than 2200 journals in topics addressing our planet’s climate emergency and journals area guides by eminent editorial boards [50]. PubMed is a free resource of the U.S. National Institutes of Health (NIH) of journals in the fields of biomedicine and life sciences and contains over 34 million citations and abstracts of biomedical literature [51]. Although the choice of five databases encompasses a broad range of the literature in the Environmental Sciences, it will certainly leave out many publications. However, as this review aims to qualitatively understand the conceptual usage of terms referring to climate change perceptions, the search strategy is considered adequate. Additionally, including databases focused only on region-specific bibliographic sources would likely bias the findings to those languages the team are able to review, possibly giving a wrong impression of regional differences.

#### Search terms and language

The search string of our review, i.e. the combination of key terms using Boolean operators (AND, OR), will include English words encompassing three groups of concepts: (i) perception or awareness; (ii) climate change or global warming; and (iii) smallholders (including indigenous population). The team will use special characters (i.e. asterisk) in the search to include alternative forms of word endings and plurals, except for BASE, Science Direct and PubMed because they do not accept asterisk. For details on the string elaboration process, see Additional file 2.

The team will review documents in English because: (i) the review team is familiar with this language and (ii) English is the universal scientific idiom. Although other languages will be excluded, the team does not believe this will bias results for three reasons. First, team intend to identify and describe the various concepts in usage and not compute every single article that used the term. Second, our pre-tests did not indicate geographical distribution in concept usage. Third, there is no direct translation of a few perception terms (e.g., attitudes, awareness) across languages and, therefore, including other languages would add inconsistencies in the coding process. Despite limiting the available evidence, this choice guarantees access to worldwide research as it reduces the selection bias that would result from choosing a small number of languages. Additionally, and more importantly, as this article aims to systematise and analyse the article’s alternative usage of the term perception, language differences would probably pose insurmountable challenges to our review, especially when definitions are implicit.

In five databases, the team will search studies with the selected terms appearing in the article’s title, abstract or keywords. The team will include common synonyms in the search terms selection (Table 1).

**Table 1:** Central terms and their synonyms, including in the string

Central terms	Synonyms included in the string	Synonyms without effect in string
Perception	perception*; local perspective*	“climate change perception”
Awareness	–	–
Climate change	climat* chang*; global warming; chang* climat*; climat* variability*; climat* event*	–
Small-scale population	indigenous; smallholder*; small scale*; livelihood*; fisher*; "peasant*"; "hunter*"; "agricultur*"; "forager*"; "agropastoralist*"; "horticultur*"; "pastoralist*”; “herder*”; “small-island*”	“indigenous group”; "hunter-gather"

The use of hyphens or not on “small scale” and “small-island” has no effect on the search. The use of asterisk includes the alternative forms of word endings and plurals

The final search string is described below in the Scopus, WoS, BASE—Bielefeld Academic Search Engine, Science Direct Elsevier and PubMed formats:

*Scopus:* (TITLE-ABS-KEY ((("perception*" OR "local perspective*" OR "awareness") AND ("climat* chang*" OR "global warming" OR "chang* climat*" OR "climat* variabilit*" OR "climat* event*") AND ("indigenous*" OR "smallholder*" OR "small scale*" OR "livelihood*" OR "fisher*" OR "peasant*" OR "hunter*" OR "agricultur*" OR "forager*" OR "agropastoralist*" OR "horticultur*" OR "pastoralist*" OR “herder*” OR “small-island*”))))

*WoS:* ALL FIELDS (("perception*" OR "local perspective*" OR "awareness") AND ("climat* chang*" OR "global warming" OR "chang* climat*" OR "climat* variabilit*" OR "climat* event*") AND ("indigenous*" OR "smallholder*" OR "small scale*" OR "livelihood*" OR "fisher*" OR "peasant*" OR "hunter*" OR "agricultur*" OR "forager*" OR "agropastoralist*" OR "horticultur*" OR "pastoralist*" OR “herder*” OR “small-island*”))

*BASE:* Entire document: ("perception" OR "awareness") AND ("climate change" OR "global warming") AND ("indigenous" OR "smallholder" OR “small-island”)

*Science Direct:* Title, abstract, keywords: (("perception" OR "awareness") AND ("climate change" OR "global warming") AND ("indigenous" OR "smallholder" OR “small-island”))

*PubMed:* (("perception" OR "awareness") AND ("climate change" OR "global warming") AND ("indigenous" OR "smallholder" OR “small-island”))

#### Estimating the comprehensiveness of the search

The comprehensiveness of the search string was tested by comparing the search result with a test list of 95 benchmark publications about climate change perception in small-scale populations. This list originated from the review team's experience with the literature on the subject, which included a previous non-systematic review. With this list, the team evaluated the percentage of articles in the Scopus, Web of Science Core Collection and BASE bibliographic databases (listed in Additional file 3). The team replaced a few terms in the string, such as: (i) removing the term “perceive*”; (ii) replacing “hunt*” with “hunter*”; (iii) replacing AND with OR; (iv) adding "small-island*”, resulting in the final string above. The final search string found 94 articles (99%) across the three databases, and we concluded that the comprehensiveness of the search strategy is sufficient.

#### Publication databases searches

We will access the five databases (Scopus, WoS Core Collection, BASE—Bielefeld Academic Search Engine, Science Direct Elsevier and PubMed) through the institutional subscription via Virtual Private Network (VPN) of the University of São Paulo in Brazil. The following two filters will be applied to the search fields in Scopus and WoS: (i) article in document type and (ii) English in language. In BASE, the team will select basic search, filtering by English in language and article contribution in document type. In Science Direct, we will select advanced search, filtering by title, abstract or author-specified keywords, and we will enter the string. In PubMed no filter will be applied. Our selection will be restricted to articles that report findings from primary data because, otherwise, they could be articles that mixed up different concepts from the original literature. The team will exclude books and book chapters because we cannot guarantee access to them. Therefore, the team will exclude the following article types from the search: (i) documents in languages other than English; (ii) review articles, books, book chapters, conference papers, proceedings papers, conference reviews, editorials, letters and data papers.

Before proceeding to the screening phase, duplicate articles from the five databases (Scopus, WoS, BASE—Bielefeld Academic Search Engine, Science Direct Elsevier and PubMed) will be identified and removed using the Excel spreadsheet. If an article was authored by someone from the review team, this person will not participate in coding decisions.

This review will not pursue additional efforts to obtain literature, such as consultation with experts or stakeholders.

### Article screening and study eligibility criteria

#### Screening process

Two successive screening stages will be followed to assess articles’ eligibility, i.e. evaluating its: (1) title and abstract, and (2) full text. Two reviewers will independently screen the title, abstract and full text, and the evaluation results will be compared. A calibration exercise will be employed for consistency checking. Articles that meet the eligibility criteria in the title and abstract will be further screened at stage two, whereas those that did not meet the inclusion criteria will be excluded at this stage. Stage two will consist of full reading papers that met the eligibility criteria in the first screening stage; otherwise, they will be excluded. At both stages, the review team will discuss in weekly meetings the discrepancies in evaluation. When disagreement persists after this meeting, the decision to include or not each article will rely on the tie-breaking vote of a third reviewer. Papers duplicated will be removed. The team will report the reasons for excluding each article in an additional file, as well as the complete record of all articles. Papers with only title and without abstract will be read in full to verify if they will be selected or not for stage two. The team will report the number of articles selected, excluded, and duplicated in each stage (stages 1 and 2) in the ROSES flow diagram [52]. The search results from each database will be exported in an Excel spreadsheet.

To ensure consistency and accuracy of inclusion/exclusion decisions throughout the screening process, two reviewers will check the process consistency through the random selection of 5% or 20 of the total sample of articles (whichever is the greater) for screening (i) title and abstract, and (ii) full text. With that sample, the team will calculate the agreement rate between reviewers regarding the list of articles that fulfilled the criteria for inclusion in stages 1 and 2. The results of the consistency checking will then be compared among reviewers, and all disagreements will be discussed in detail until the consistency level reaches at least 80%.

#### Eligibility criteria

To be included in our systematic map, studies must meet the following criteria:

- Population: the team will include articles that deal only with small-scale populations (including indigenous people) inhabiting rural areas. Therefore, the team will exclude articles dealing with extensive rural properties, such as those dedicated to commercial monoculture directed towards commodity markets (e.g., agribusiness). Eligible small-scale populations include hunter-gatherers or foragers, mainly or at least partially subsistence-based family farmers or horticulturists, fishers using small and medium-sized wooden boats, pastoralists or herders, and family agropastoralists. Studies that deal with small-scale populations that moved to urban areas will be discarded, as will be those cases in which there is no specification of the target population.
- Outcomes: the article should address the concept of climate change perception, regardless of whether its definition will be presented explicitly or implicitly.
- Types of study design: the team will consider empirical studies based on primary data, employing quantitative or/and qualitative methods of data gathering.

The list of articles excluded at the full-text screening stage will be listed in an additional file together with the reasons for their exclusion.

#### Study validity assessment

Critical appraisal of the validity and quality of studies is optional in systematic mapping and should be adopted only when there is a sufficient level of methodological details, such as necessary to evaluate the external and internal validity of studies [45]. A process causality or its generalisation to specific contexts is irrelevant to our research. Instead, the team are interested in describing the variability in concept usage across studies to get an overview of the conceptual similarities and differences in the climate change literature. Because of that, we also do not intend to restrict our choice of eligible articles.

### Data coding strategy

To check for intercoder consistency, two team members will independently code a sample of 1% of the total number of articles. The selection of these articles in the databases will follow the sort order by date in the output list, i.e. the team will read first the articles with the most recent dates appearing at the top of the list. After coding this article’s sample, the review team will discuss inconsistencies and doubts. Decisions on disagreements will then be taken, when necessary, with the help of a third team member.

When coding, all the eligible articles will be double-screened to ensure consistency. Weekly team meetings will be arranged to discuss problems and align activities between reviewers to guarantee coding consistency.

While conducting full-text screening (described above), the team will perform data extraction and coding but will keep it only for those texts that meet the inclusion criteria. Data coding will rely on a codebook prepared before mapping begins (Additional file 4). Missing or unclear data will be specified in the process. The team will use the following structure for data extraction and coding for articles that met the eligibility criteria and were then selected for full reading. For more details of the items below, see Additional file 4.

- Bibliographic information: title, authors, journal, year, DOI.
- Study location: a general description of the country.
- Origin of the investigated population: all indigenous, all non-indigenous, mixed indigenous and non-indigenous, non-specified.
- Subsistence strategy of the investigated population: small-scale agriculturalist, small-scale agropastoralist, forager or hunter-gatherer or fisher-gatherer, horticulturalists, pastoralist or herder, other.
- Thematic areas covered in the article: adaptation, awareness, traditional knowledge with and without indicators, scientific knowledge, comparison between observations of individual perceptions with scientific data, observed changes in livelihood activities, observed changes in the environment, determining factors (i.e. perception drivers), mitigation, resilience, risk perception, another thematic area.
- Existence or nonexistence of explicit perception definition: explicit, implicit, other.
- Part of the text where the perception definition appears: introduction, methods, results, discussion, conclusion, other.
- Description of the explicit definition of perception of climate change: as described by the authors.
- Description of which construct(s) is(are) part of the climate change perception definition(s) adopted in the article, meaning that perception is measured, for instance, as the observation of changes in the environment; as a sensory experience; as people’s belief that a climate change is occurring, attitudes, traditional knowledge, among others.
- Description of how perception is presented in the article’s results, discussion or conclusion: regardless of whether or not there is a definition of perception, for instance, perception is described as the observation of changes, perception is defined as traditional or scientific knowledge, or as the individual's degree of concern about the effects of climate change on livelihood activities, among others.
- Description of how perception appears in the results: as described by the authors (when explicit).
- Description of the phenomenon observed in the article: physical phenomenon, biological phenomenon, human phenomenon, other phenomenon.
- Description of the sampling unit of collected data in methods: individual level, household level, individual and household level, community level or village, organisations (e.g., NGOs, associations), others.
- Description of data used in the article to analyse methods: primary data, secondary data, primary and secondary data, others.
- Description of methods used in the article: qualitative methods, quantitative methods, mixed methods, others.

### Data mapping method

The team will produce a database with the information extracted from the articles in Excel spreadsheet format. The complete worksheet will be made accessible in Additional files, including a catalogue with all definitions of perception identified by the reviewers.

The team intend to summarise the relevant results in a narrative synthesis and adopt analytical (e.g., descriptive statistics) and visualisation tools (e.g., maps tables, graphs and other figures) to present the mapping results, such as the following examples.

- Table: a synthesis with the description of the several concepts adopted in the literature, together with examples of the relevant studies.
- Graph—Temporal: describing the temporal trends in the frequency of definition usage.
- Figure—Evidence atlas: describing the location of all studies across the geographical space and subdivided in concept type if there are regional differences.
- Graph—Heat map: describing the usage of the alternative perception definitions (x-axis) by subsistence strategy (y-axis).
- Graph—Heat map: the construct of perception definitions (x-axis), by observation of phenomenon (y-axis).
- Graph—Two-dimensional categorial bubble plots: the origin of the investigated population (x-axis), by subsistence strategy of the surveyed population (y-axis).
- Graph—Two-dimensional categorial bubble plots: the origin of the investigated population (x-axis), by thematic areas covered in the article (y-axis).
- Graph—Two-dimensional categorial bubble plots: sampling unit (x-axis), by perception description in the results, discussion or conclusion (y-axis).
- Graph—Two-dimensional categorial bubble plots: continent (x-axis), by the origin of the investigated population (y-axis).

The presentation of crucial information will depend on the articles that pass the screening stage but will presumably contain information based on the Codebook.

A data file of all screened literature, with the reason for inclusion and exclusion and associated metadata will also be made available upon completion.

## Supplementary Information

**Additional file 1. **ROSES checklist of systematic map protocol.

**Additional file 2.** Search string development.

**Additional file 3.** List of the 95 articles used to assess the comprehensiveness of the search string.

**Additional file ﻿4.** Codebook—Description of the data that will be extracted in the systematic map.

## Data Availability

Not applicable.
