# Supplementary material for: What evidence exists on conceptual differences in climate change perceptions of smallholders? A systematic map protocol
Source: Environ Evid. 2022 Sep 16;11:31. doi: 10.1186/s13750-022-00284-w (PMC11378815; doi:10.1186/s13750-022-00284-w)
Supplement: Supplementary file 2 — Additional file 2. Search string development. [file 13750_2022_284_MOESM2_ESM.docx]

**Additional file 2: Search string development.**

This document summarizes the main steps taken to identify search terms and develop the search string. For this, we defined a set of keywords by consulting scientific articles in the area of climate change. For the constructing exercise to build the search string, we performed searches on the Web of Science Core Collection (WoS) and Scopus databases on Topic (TS). We built the search string through several steps detailed below, starting on 04/30/2021 and ending on 07/11/2022, in the preparation of the protocol.

As described in our protocol, we use Boolean operators, alternate terms, and asterisks. We used the asterisk to allow the identification of singular or plural words in the same search. We compiled our search strings using the following key terms: perception, awareness, climate change, and small-scale population. We separated the key terms by parentheses and use quotation marks in every word.

Search String nº 1 and nº 2

We started string testing in Scopus and WoS on April 2021. This was the first search string:

*("climate change" OR "environmental change") AND ("perception*" OR "perceived" OR "awareness") AND ("indigenous people*" OR "smallholder" OR "small group" OR "indigenous group")*

The result of the first test resulted in 483 publications in Scopus and 517 in WoS, a number considered low compared to the wide coverage that both databases have. Therefore, we decided to redo the tests.

After the first test of the string, we started testing which terms made a difference or not in the results. We saw that the term "indigenous group" could be dropped, resulting in 516 publications in Scopus and 482 in WoS. This was the second search string:

*("climate change" OR "environmental change") AND ("perception*" OR "perceived" OR "awareness") AND ("indigenous people*" OR "smallholder" OR "small group")*

Search String nº 3 and nº 4

As we are interested in small-scale populations, we decided to insert the term “small scale” in our string, which resulted in 666 publications in Scopus and 720 in WoS. We observed that the hyphen in "small scale" did not make a difference in the string, because of this we chose to leave it without a hyphen.

*("climate change" OR "environmental change") AND ("perception*" OR "perceived" OR "awareness") AND ("indigenous people*" OR "smallholder" OR "small group" OR “small scale")*

We also added the asterisk in the term "small group" and in "small scale" so that it also represented both words in the plural. This sequence increased by one publication in Scopus (N=667) and 9 in WoS (N=729), resulting in the following string:

*("climate change" OR "environmental change") AND ("perception*" OR "perceived" OR "awareness") AND ("indigenous people*" OR "smallholder" OR "small group*" OR “small scale*")*

We saw that changing the order of groups of terms in the string (i.e. *("perception*"OR “perceived" OR "awareness") AND ("climate change" OR "environmental change") AND ("indigenous people*" OR "smallholder" OR "small group*" OR “small scale*")*) does not influence the final search result.

Search String nº 5 and nº 6

We chose to add the term "global warming", resulting in 684 publications in Scopus and 741 in WoS.

*("perception*" OR "perceived" OR "awareness") AND ("climate change" OR "environmental change" OR “global warming”) AND ("indigenous people*" OR "smallholder" OR "small group*" OR “small scale*")*

The term "smallholder" proved to be important in the string, because when we excluded it, the number of publications decreased to 364 in Scopus and 371 in WoS. Therefore, we chose to keep it in the string and added asterisk, resulting in 685 publications in Scopus and 781 in WoS. Adding the term "climate change perception" had no effect on the increase in publications, so it was not included in the string. In order to increase the scope of the results for words that could be written in the plural, we put the asterisk in “climate change” and “environmental change”. The change returned 692 publications in Scopus and 810 in WoS and the string as follow below:

*("perception*" OR "perceived" OR "awareness") AND ("climat* chang*" OR "environment* chang*" OR “global warming”) AND ("indigenous people*" OR “smallholder*” OR "small group*" OR “small scale*")*

Search String nº 7 and nº 8

In addition to the term "climat* chang*, we chose to include first the term "chang* climat*", resulting in 697 publications in Scopus and 819 in WoS.

*("perception*" OR "perceived" OR "awareness") AND ("climat* chang*" OR "environment* chang*" OR “global warming” OR “chang* climat*”) AND ("indigenous people*" OR “smallholder*” OR "small group*" OR “small scale*")*

We also chose to insert the term “climat* variability”, returning 721 publications in Scopus and 842 in WoS.

*("perception*" OR "perceived" OR "awareness") AND ("climat* chang*" OR "environment* chang*" OR “global warming” OR “chang* climat*” OR “climat* variability”) AND ("indigenous people*" OR “smallholder*” OR "small group*" OR “small scale*")*

Search String nº 9 and nº 10

When we added the term "rural" the searches increased (1,667 publications in Scopus and 2,222 in WoS). The increase was also verified when we entered "livelihood*" (2,263 publications in Scopus and 2,816 publications in WoS), according to the string below:

*("perception*" OR "perceived" OR "awareness") AND ("climat* chang*" OR "environment* chang*" OR “global warming” OR “chang* climat*” OR “climat* variability”) AND ("indigenous people*" OR “smallholder*” OR "small group*" OR “small scale*" OR “rural” OR “livelihood*”)*

We added the term "indigenous" without the word people, resulting in 2,472 publications in Scopus and 3,037 in WoS.

*("perception*" OR "perceived" OR "awareness") AND ("climat* chang*" OR "environment* chang*" OR “global warming” OR “chang* climat*” OR “climat* variability”) AND ("indigenous people*" OR “indigenous” OR “smallholder*” OR "small group*" OR “small scale*" OR “rural” OR “livelihood*”)*

Search String nº 11 and nº 12

We changed the term "perceived" to "perceive" and added “local perspective*” and “climat* event*”, returning 2,570 publications in Scopus and 3,122 publications in WoS, as follow below:

*("perception*" OR "perceive*" OR “local perspective*” OR "awareness") AND ("climat* chang*" OR "environment* chang*" OR “global warming” OR “chang* climat*” OR “climat* variability” OR “climat* event*”) AND ("indigenous people*" OR “smallholder*” OR "small group*" OR “small scale*" OR “rural” OR “livelihood*”)*

When we added the term "farm*", the search results were for 3,604 in Scopus and 4,169 in WoS, according to the following string:

*("perception*" OR "perceive*" OR “local perspective*” OR "awareness") AND ("climat* chang*" OR "environment* chang*" OR “global warming” OR “chang* climat*” OR “climat* variability” OR “climat* event*”) AND ("indigenous people*" OR “smallholder*” OR "small group*" OR “small scale*" OR “rural” OR “livelihood*” OR “farm*”)*

Search String nº 13 and nº 14

We deleted the term "environment* chang*" as it involves changes other than climate change, and we chose to remove the term "indigenous people*", as we understand that term "indigenous" is sufficient. Added asterisk in “climat* variabilit*” to include more publications (3,477 publications in Scopus and 4,109 in WoS).

*(“perception*” OR “perceive*” OR “local perspective*” OR “awareness”) AND (“climat* chang*” OR “global warming” OR “chang* climat*” OR “climat* variabilit*” OR “climat* event*”) AND (“indigenous*” OR “smallholder*” OR “small group*” OR “small scale*” OR “rural” OR “livelihood*” OR “farm*”)*

To further specify our search, we replaced the terms “rural” and “farm*” with “subsistence”, as we are interested in small-scale populations that engage in subsistence activities. In addition, we added more details about this population of interest, with the terms "fisher*", "peasant*", "hunt*", "agricultur*", "forager*", "hunter-gather", "gather*", "agropastoralist*", "horticultur*", "pastoralist*".

*("perception*" OR "perceive*" OR "local perspective*" OR "awareness") AND ("climat* chang*" OR "global warming" OR "chang* climat*" OR "climat* variabilit*" OR "climat* event*") AND ("indigenous*" OR "smallholder*" OR "small group*" OR "small scale*" OR "livelihood*" OR "subsistence") AND ("fisher*" OR "peasant*" OR "hunt*" OR "agricultur*" OR "forager*" OR "hunter-gather" OR "gather*" OR "agropastoralist*"  OR  "horticultur*"  OR  "pastoralist*")*

Search String nº 15 and nº 16

We excluded the term "small group", as it was returning posts with other meanings, such as small group of smartphone, small group of users, small group discussion, small-group deliberations, small-group sea level rise discussions or small group of residents. Furthermore, we excluded the terms “subsistence” and “gather*”. Our searches result in 924 publications in Scopus and 1,197 in WoS. We saw that "hunter-gather" term had no effect on the results, the search resulted in the same number of publications in both databases, so we excluded the term.

*("perception*" OR "perceive*" OR "local perspective*" OR "awareness") AND ("climat* chang*" OR "global warming" OR "chang* climat*" OR "climat* variabilit*" OR "climat* event*") AND ("indigenous*" OR "smallholder*" OR "small scale*" OR "livelihood*") AND ("fisher*" OR "peasant*" OR "hunt*" OR "agricultur*" OR "forager*" OR "agropastoralist*"  OR  "horticultur*"  OR  "pastoralist*")*

When looking to improve the string, we deleted the term "perceive*", changed the term "hunt*" to "hunter*" and added "herder*". The search resulted in 856 publications in Scopus and 1,126 in WoS.

*("perception*" OR "local perspective*" OR "awareness") AND ("climat* chang*" OR "global warming" OR "chang* climat*" OR "climat* variabilit*" OR "climat* event*") AND ("indigenous*" OR "smallholder*" OR "small scale*" OR "livelihood*") AND ("fisher*" OR "peasant*" OR "hunter*" OR "agricultur*" OR "forager*" OR "agropastoralist*"  OR  "horticultur*"  OR  "pastoralist*" OR “herder*”)*

Search String nº 17

We realized that we should replace the Boolean character OR with AND, since we understood that the terms used were synonymous for small-scale population. Therefore, this was the string:

*("perception*" OR "local perspective*" OR "awareness") AND ("climat* chang*" OR "global warming" OR "chang* climat*" OR "climat* variabilit*" OR "climat* event*") AND ("indigenous*" OR "smallholder*" OR "small scale*" OR "livelihood*" OR "fisher*" OR "peasant*" OR "hunter*" OR "agricultur*" OR "forager*" OR "agropastoralist*"  OR  "horticultur*"  OR  "pastoralist*" OR “herder*”)*

As of May 2022, the search returned 3,695 publications in Scopus and 4,343 publications in WoS. When we apply the filter for articles in document type, we have 2,842 publications in Scopus and 3,813 in WoS. Further refining the search for English language and article in document type, we have 2,764 publications in Scopus and 3,773 in WoS.

Search String nº 18 (final string)

In July 2022, we realized that we did not include small-scale islanders. We added the term "small-island" to our string, which resulted in the final string:

*("perception*" OR "local perspective*" OR "awareness") AND ("climat* chang*" OR "global warming" OR "chang* climat*" OR "climat* variabilit*" OR "climat* event*") AND ("indigenous*" OR "smallholder*" OR "small scale*" OR "livelihood*" OR "fisher*" OR "peasant*" OR "hunter*" OR "agricultur*" OR "forager*" OR "agropastoralist*"  OR  "horticultur*"  OR  "pastoralist*" OR “herder*” OR “small-island*”)*

We refined the search for English language and article in document type, we have 2,886 publications in Scopus and 3,952 in WoS.

Additional comments

We adapted the search string for the BASE - Bielefeld Academic Search Engine, Science Direct Elsevier and PubMed databases, as it did not cover the same number of characters as Scopus and WoS. Therefore, the final string for the BASE, Science Direct Elservier, and PubMed was:

(("perception" OR "awareness") AND ("climate change" OR "global warming") AND ("indigenous" OR "smallholder" OR “small-island”))
