# Supplementary material for: What evidence exists on conceptual differences in climate change perceptions of smallholders? A systematic map protocol
Source: Environ Evid. 2022 Sep 16;11:31. doi: 10.1186/s13750-022-00284-w (PMC11378815; doi:10.1186/s13750-022-00284-w)
Supplement: Supplementary file 4 — Additional file ﻿4. Codebook—Description of the data that will be extracted in the systematic map. [file 13750_2022_284_MOESM4_ESM.docx]

**Additional File 4: Codebook - Description the data extracted for the systematic map.**

**CODEBOOK**

A. BIBLIOGRAPHIC INFORMATION

- **Article ID**: a code number or combination of numbers (i.e., XXXX+YYYY), where XXXX is the current number as 0001, 0002,…,000n and YYYY is the year (e.g., 2020). For example, 00012020
- **Article source:**

1 = WoS Core Collection

2 = Scopus

3 = BASE

4 = Science Direct

5 = PubMed

6 = WoS and Scopus

7 = WoS and BASE

8 = WoS and Science Direct

9 = WoS and PubMed

10 = WoS, Scopus, BASE

11 = WoS, Scopus, Science Direct

12 = WoS, Scopus, PubMed

13= WoS, BASE, Science Direct

14 = WoS, BASE, PubMed

15 = WoS, Science Direct, PubMed

16= Scopus and BASE

17= Scopus and Science Direct

18= Scopus and PubMed

19= Scopus, BASE, Science Direct

20 = Scopus, Science Direct, PubMed

21 = Scopus, BASE, PubMed

22 = BASE and Science Direct

23 = BASE and PubMed

24 = BASE, Science Direct, PubMed

25 = Science Direct and PubMed

26 = WoS, Scopus, BASE, Science Direct

27 = WoS, Scopus, BASE, PubMed

28 = WoS, Scopus, Science Direct, PubMed

29 = WoS, Scopus, BASE, Science Direct, PubMed (all databases)

- **Article title**: full text, e.g., “Determinants of choice of climate change adaptation practices by smallholder pineapple farmers in the semi-deciduous forest zone of Ghana”.
- **Author(s):** text, e.g. Antwi-Agyei, P.; Wiafe, E.A.; Amanor, K.; Baffour-Ata, F.; Codjoe, S.N.A.
- **Journal:** text, e.g. Environmental and Sustainability Indicators
- **Year**: number (YYYY), e.g. 2020
- **DOI:** an alphanumeric string of characters, e.g., 10.1016/j.jenvman.2021.113801

B. STUDY GENERAL DESCRIPTION

- **Country number:** How many countries are described in the article?

1 = **One country:** the study was carried out only in one country.

2 = **Two countries**: the study was carried out in two countries.

3 = **Three countries**: the study was carried out in three countries.

4 = **Four countries**: the study was carried out in four countries.

5 = **More than five countries:** the study was carried out in more than five countries.

6 = **Others above five.**

- **Country one**: number (a combination of number X+YY, where X is the continent and YY refers to a country). From the list below, select only the first country.
- **Country two:** number (a combination of number X+YY, where X is the continent and YY is a country). From the list below, select only the second country. If not, write 0 (0=not applicable).
- **Country three:** number (a combination of number X+YY, where X is the continent and YY is a country). From the list below, select only the third country. If not, write 0 (0=not applicable).
- **Country four:** number (a combination of number X+YY, where X is the continent and YY is a country). From the list below, select only the fourth country. If not, write 0 (0=not applicable).
- **Country five:** number (a combination of number X+YY, where X is the continent and YY is a country). From the list below, select only the fifth country. If not, write 0 (0=not applicable).

List of countries:

0 = Not applicable.

1 = Africa

101 = Algeria

102 = Angola

103 = Benin

104 = Botswana

105 = Burkina Faso

106 = Burundi

107 = Cabo Verde/Cape Verde

108 = Cameroon

109 = Central African Republic (the)

110 = Chad

111 = Comoros (the)

112 = Congo/Republic of the Congo

113 = Democratic Republic of the Congo

114 = Djibouti

115 = Egypt (transcontinental - generally considered African)

116 = Equatorial Guinea

117 = Eritrea

118 = Eswatini (formerly Swaziland)

119 = Ethiopia

120 = Gabon

121 = Gambia, The

122 = Ghana

123 = Guinea

124 = Guinea-Bissau

125 = Ivory Coast/Republic of Côte d'Ivoire

126 = Kenya

127 = Lesotho

128 = Liberia

129 = Libya

130 = Madagascar

131 = Malawi

132 = Mali

133 = Mauritania

134 = Mauritius

135 = Morocco

136 = Mozambique

137 = Namibia

138 = Niger

139 = Nigeria

140 = Rwanda

141 = Sao Tome and Principe

142 = Senegal

143 = Seychelles

144 = Sierra Leone

145 = Somalia

146 = South Africa

147 = South Sudan

148 = Sudan

149 = Tanzania

150 = Togo

151 = Tunisia

152 = Uganda

153 = Zambia

154 = Zimbabwe

2 = Asia

201 = Afghanistan

202 = Armenia (transcontinental - generally considered Asian)

203 = Azerbaijan (transcontinental - generally considered Asian)

204 = Bahrain

205 = Bangladesh

206 = Bhutan

207 = British Indian Ocean Territory (UK territory)

208 = Brunei Darussalam

209 = Cambodia

210 = China

211 = Hong Kong (China - Special Administrative Region)

212 = India

213 = Indonesia

214 = Iran

215 = Iraq

216 = Israel (U.N. member, though partially unrecognized)

217 = Japan

218 = Jordan

219 = Kazakhstan (transcontinental - generally considered Asian)

220 = Kuwait

221 = Kyrgyzstan

222 = Laos

223 = Lebanon

224 = Macau (China - Special Administrative Region)

225 = Malaysia

226. = Maldives

227 = Mongolia

228 = Myanmar (formerly Burma)

229 = Nepal

230 = North Korea (Democratic People's Republic of Korea)

231 = Oman

232 = Pakistan

233 = Palestine (limited recognition)

234 = Philippines

235 = Qatar

236 = Saudi Arabia

237 = Singapore

238 = South Korea (Republic of Korea)

239 = Sri Lanka

240 = Syria

241 = Taiwan (limited recognition - claimed by China)

242 = Tajikistan

243 = Thailand

244 = Timor-Leste/East Timor

245 = Turkey (transcontinental - generally considered European)

246 = Turkmenistan

247 = United Arab Emirates

248 = Uzbekistan

249 = Vietnam

250 = Yemen

3 Europe

301 = Albania

302 = Andorra

303 = Austria

304 = Belarus

305 = Belgium

306 = Bosnia and Herzegovina

307 = Bulgaria

308 = Croatia

309 = Cyprus (generally considered European)

310 = Czechia/Czech Republic

311 = Denmark

312 = Estonia

313 = Finland

314 = France

315 = Georgia (transcontinental)

316 = Germany

317 = Greece

318 = Hungary

319 = Iceland

320 = Ireland

321 = Italy

322 = Latvia

323 = Liechtenstein

324 = Lithuania

325 = Luxembourg

326 = Malta

327 = Moldova (Republic of Moldova)

328 = Monaco

329 = Montenegro

330 = Netherlands

331 = North Macedonia

332 = Norway

333 = Poland

334 = Portugal

335 = Romania

336 = Russia (transcontinental - generally considered European)

337 = San Marino

338 = Serbia

339 = Slovakia

340 = Slovenia

341 = Spain

342 = Sweden

343 = Switzerland

344 = Ukraine

345 = United Kingdom

346 = Vatican City (Holy See)*

4 = North America

401 = Antigua and Barbuda

402 = Bahamas

403 = Barbados

404 = Belize

405 = Canada

406 = Costa Rica

407 = Cuba

408 = Dominica

409 = Dominican Republic

410 = El Salvador

411 = Grenada

412 = Guatemala

413 = Haiti

414 = Honduras

415 = Jamaica

416 = Mexico

417 = Nicaragua

418 = Panama

419 = Saint Kitts and Nevis

420 = Saint Lucia

421 = Saint Vincent and the Grenadines

422 = Trinidad and Tobago

423 = United States of America

5 = Oceania

501 = Australia

502 = Fiji

503 = Kiribati

504 = Marshall Islands

505 = Micronesia (The Federated States of)

506 = Nauru

507 = New Zealand

508 = Palau

509 = Papua New Guinea

510 = Samoa

511 = Solomon Islands

512 = Tonga

513 = Tuvalu

514 = Vanuatu

6 = South America

601 = Argentina

602 = Bolivia

603 = Brazil

604 = Chile

605 = Colombia

606 = Ecuador

607 = Guyana

608 = Paraguay

609 = Peru

610 = Suriname

611 = Uruguay

612 = Venezuela

7 = Unknown

8 = Another country

C. POPULATION DESCRIPTION

- **Origin of the investigated population:** choose the option that best describes the population investigated in the article.

1 = **All indigenous.** We will consider as indigenous those inhabitants of rural (non-urban) areas, entitled in the article as indigenous, native, native people, autochthonous, aboriginal, even if described in the article as an emerging society (i.e., when they reassume their indigenous identity after some time). Indigenous peoples are considered those that: (i) share inter-generational ancestry and cultural aspects with original (pre-colonial or pre-invasion) occupants of ancestral lands in a specific region of the world; (ii) consider themselves distinct from other sectors of the current prevailing societies; (iii) usually speak a different language; (iv) have diverse cultures and worldviews; (v) often aim to preserve, develop, and transmit to future generations their ancestral territories and ethnic identities, as the basis of their continued existence as a society; (vi) have and are determined to maintain their own cultural patterns, social institutions, and common-law system; (vii) often share deep social, cultural, and spiritual ties to their ‘‘lands’’ (a term that captures Indigenous territories in general, including terrestrial, water, and associated spiritual environments), and (viii) their livelihoods, health, and well-being are closely linked to activities such as hunting, fishing, herding, foraging, small-scale family farming, and land and/or water management practices that have developed over many generations.

0 = **All non-indigenous.** We will consider small-scale populations living in rural (non-urban) areas as non-indigenous, when: (i) entitled in the article as a farmer, peasant, smallholder, small-scale fisher, small-scale hunter, and (ii) not accompanied by terms such as an indigenous person, native, autochthonous, native people, aboriginal, among others.

2 = **Mixed indigenous and non-indigenous**. We will consider mixed when the article deals with the two types of populations (indigenous and non-indigenous), whether these populations belong to the same place or from two or more different locations.

3 = **Not specified in the text.**

4 = **Another specified origin, but not listed above.**

- **Subsistence strategy of the investigated population:**

Rule: consider, in this order, (i) the subsistence activity described as the main one in the article; (ii) when not classified or highlighted, choose the most frequent/most often performed activity (in time); (iii) if missing, choose the activity that people allocate more time to; (iv) when missing, consider the activity that generates the most significant percentage of income (total income and, if absent, monetary income).

1 = **Small-scale** **Agriculturalist** are individuals who: (i) practice agriculture mainly to their subsistence; (ii) practice land use involving crop rotation or permanent cultivation in the same year or for more years; (iii) may involve labour beyond the nuclear family; (iv) may use tools (mainly manual), but do not rely on mechanisation; (v) they can sell part of their production on the market as a complement to their income. Agriculture based on monocultures directed to agribusinesses are excluded.

2 = **Small-scale Agropastoralists** rely mainly on agriculture and pastoralism for their subsistence. They might sell part of their agricultural or animal production to raise some cash, but the output is not directed only nor mainly to markets.

3 = **Forager, Hunter-Gatherer or Fisher-Gatherer** are individuals who: (i) practice subsistence-based on HUNTING wild animals, FISHING, and GATHERING wild plants as their primary subsistence strategy; (ii) they do not rely on plant or animal domestication; (iii) division of labour tends to be by age and gender; (iv) often live in small groups (band-organised), which may concentrate in larger groups and, subsequently, disperse; (v) are currently or used to be nomadic in natural landscapes; (vi) their lands tenure is often a common property regime (a kinship-based collective holds land with rules of reciprocal access); (vii) sharing and reciprocity are central rules of their social interaction; (viii) are more egalitarian.

31 = **Fisher-Gatherer.** Foragers depend mainly on fishing for their subsistence and as a source of protein.

32 = **Hunter-Gatherer.** Foragers depend mainly on hunting for their subsistence and as a source of protein.

4 = **Horticulturists** are individuals who: (i) are small-scale (often family) farmers who produce mainly for their consumption (i.e., subsistence farmers) but may trade the surplus of their production; (ii) depend on domesticated plants and trees for the bulk of their food energy; (iii) may raise a few animals for their consumption (or as saving); (iv) gender division of labour is standard, and labour is provided by the producer's nuclear or extended family; (v) form permanent settlements with a few dozens to several hundred people in well-defined villages or clan territories; (vi) fields are often used for only a couple of years and then allowed to fallow 2 to 15 years; (vii) generally, practice shifting cultivation; (viii) often, but not only, practice slash-and-burn agriculture; (ix) farming is often practised together with foraging activities; (x) use hand tools to cultivate.

41 = **Horticulturalist with fishing.** Horticulturists who depend mainly on fishing as a source of protein (subsistence).

42 = **Horticulturalist with hunting**. Horticulturists who depend mainly on hunting as a source of protein (subsistence).

5 = **Pastoralists or herders** are individuals who: (i) herd animals as their primary source of subsistence (e.g. [cattle](https://en.wikipedia.org/wiki/Cattle), [camels](https://en.wikipedia.org/wiki/Camel), sheep, [goats](https://en.wikipedia.org/wiki/Goat), llamas, [yaks](https://en.wikipedia.org/wiki/Yak), [reindeer](https://en.wikipedia.org/wiki/Reindeer), [horse](https://en.wikipedia.org/wiki/Horse)s) found in various parts of the world; (ii) use animals not only as food (e.g. meat, milk, cheese, butter), but also for fur, bones and as beasts of burden; (iii) often sell some animals or animal products to raise cash, as a complementary strategy; (iv) land use is extensive with animals moved to pasture instead of fodder brought to them; (v) may practice agriculture for own consumption or to feed animals, but this is not how they spend most of their time (often seasonally); (vi) rely on the gender division of labour; (vii) there are sedentary pastoralists, but most of them are nomadic, moving seasonally or temporarily to pastures as needed; (viii) rely on a concept of ownership restricted to animals, housing and some domestic goods, but not land.

6 = **Another specified subsistence category, but not listed above.**

7 = **Non-specified.**

8 =**Fishers** are individuals who depend mainly on fishing for their subsistence and as a source of protein. Consider fishers, when the only description of the activity performed is fishing activity.

D. OUTCOME DESCRIPTION

- **Definition form:** how the perception definition appears in the text.

1 = **Explicit:** when definitions are explicitly written, i.e., with a direct explanation in the text in alternative forms such as perception (or any other construct) is, perception equals, local perception means, among others.

0 = **Implicit:** when definitions are not explicitly written, i.e., the article does not contain a description of the perception concept, but refers to it indirectly (i.e., do not use “perception—or any other equivalent construct— is, perception equals, local perception means”, among others). Consider as implicit when it appears, for instance, only how it was measured in the methods or results.

2 = **Another.**

- **Definition where:** part of the text where the definition appears**.**

0 = Not applicable.

1 = Introduction

2 = Methods

3 = Results

4 = Discussion

5 = Conclusion

6 = Another part not specified.

- **Definition term adopted:** text or n⁄a (not applicable). Copy exactly the sentence that contains the explicit definition of perception. The definition may be taken from other authors. For example, the article is from “Cardoso et al., 2020”, but the definition is from "Barbosa et al., 1996". Include a note on this.
- **Perception as a construct:** Is the perception construct part of the definition of perception? 0=No; 1=Yes. Consider perception “the set of processes by which we recognise, organise, and make sense of the sensations we receive from environmental stimuli” (Sternberg; Sternberg; Mio, 2012, p. 535). Perception includes sensory experience, including sight, hearing, touch, taste, smell, and other stimuli received from the environment. The person interprets the stimuli into something meaningful based on prior experiences, knowledge and subjective interpretations of reality (Pike; Edgar; Edgar, 2012; Sternberg; Sternberg; Mio, 2012). Sensory experiences include observation.
- **Awareness as a construct:** Is the awareness construct part of the definition of perception? 0=No; 1=Yes. Consider awareness in the context of climate change (CC) as “the process to perceive and understand CC as a hazard as well as to increase the willingness of taking action in a committed and collaborative way to adapt and face the challenges of CC" (Iturriza et al., 2020, p.3). Awareness of climate change comprises the perception of climate change as a problem and threat (Arlt; Hoppe; Wolling, 2011).
- **Consciousness as a construct:** Is the consciousness construct part of the definition of perception? 0=No; 1=Yes. The concept of consciousness in cognitive psychology “encompasses the state of being awake, our ability to control our behaviour and be aware of our surroundings, and our mental experiences” (Andrade, 2012, p. 583). Consciousness consists of a multidimensional set of psychological constructs such as beliefs, attitudes, knowledge, values and taking action (behaviour) (Sánchez & Lafuente, 2010; Triantafyllidou & Zabaniotou, 2021). Synonyms of consciousness are environmental concern or environmental consciousness (Sánchez & Lafuente, 2010). Environmental consciousness can lead to pro-environmental behaviour in climate change, i.e., individuals who have an environmental consciousness can take actions that mitigate, minimise, or avoid the adverse impacts of climate change.
- **Attitude as a construct:** Is the attitude construct part of the definition of perception? 0=No; 1=Yes. Consider attitude “a psychological tendency that is expressed by evaluating a particular entity with some degree of favour or disfavour” (Chaiken & Eagly, 1993, p.1) towards climate change. “Attitudes have a subject matter, which can be an object, a person, or an abstract idea” (Albarracin & Shavitt, 2018, p. 300).
- **Belief as a construct:** Is the belief construct part of the definition of perception? 0=No; 1=Yes. Consider belief as “a personal conviction that is not necessarily supported by science-based evidence - but that is shaped by the overall context in which they occur, including the scientific understanding we have of it” (Sousa-Silva et al., 2016, p.2). Aspects learned from experience or at school, or even misconceptions and incomplete truths people believe are valid beliefs (Ardoin; Heimlich; Braus & Merrick, 2013). For example, people perceive climate change processes as a threat or natural phenomenon despite no scientific knowledge.
- **Concern as a construct:** Is the concern construct part of the definition of perception? 0=No; 1=Yes. Consider concern as a feeling of worry about the outcomes and consequences of climate change (Poortinga et al., 2019).
- **Direct experience construct:** Is the direct experience construct part of the definition of perception? 0=No; 1=Yes. Consider direct experience as experiencing, observing, having contact with effects of climate change directly, i.e. the individual is directly exposed, has a real adventure, is personally experiencing or observing signals of climate change situation such as heatwaves, changes to seasons, warmer winters or summers, less rain, milder winters, less snow, change to animal or plant species (Akerlof et al. 2013).
- **Indirect experience construct:** Is an indirect experience part of the perception definition? 0=No; 1=Yes. Consider as indirect experience as experiencing, observing, having contact with the effects of climate change indirectly, i.e. the individual is physically distant from the climate change situation and maybe listening about, reading or visualising the phenomenon in another person/location (Hamilton-Webb et al., 2017). Indirect experience may include: (i) observing another individual and feeling what he feels, through second or third-hand accounts from people known or unknown (vicarious experience) (Paton et al., 2000), and (ii) experience the phenomenon through media coverage, newspapers, television, internet (virtual experience).
- **Scientific knowledge construct:** Is the scientific knowledge construct part of the definition of perception? 0=No; 1=Yes. Consider scientific knowledge “usually referring to the often explicit knowledge that has been derived from applying more formal methods that aim to increase rigour in relation to different positions on validity and reliability. This includes natural science and social science research” (Raymond et al., 2010, p. 1769).
- **Traditional or indigenous or local knowledge construct:** Is the traditional knowledge construct part of the definition of perception? 0=No; 1=Yes. Consider traditional knowledge “the knowledge, innovations, and practices of both indigenous and local communities around the world that are deeply grounded in history and experience” (IPCC, 2014, p.1774). Therefore, the knowledge that: (i) tends to be the result of cumulative experience and observation, tested in the context of everyday life; (ii) devolved by oral communication and repetitive engagement rather than through formal instruction; (iii) dynamic and adapted to cultural and environmental changes, and (iv) generally transmitted orally from generation to generation (IPCC, 2014).
- **Risk perception construct:** Is the risk perception construct part of the definition of perception? 0=No; 1=Yes. Consider risk perception the “subjective judgment that people make about the characteristics and severity of a risk” (IPCC, 2014, p.1772), in this case, risks associated with climate changes. Risk is often represented as the “probability of occurrence of hazardous events or trends multiplied by the impacts if these events or trends occur. Risk of a certain person or community results from the interaction of its vulnerability, exposure, and hazard associated with climate change” (IPCC, 2014, p.1772).
- **Worldview construct:** Is the worldview construct part of the definition of perception? 0=No; 1=Yes. A worldview implies a coherent and broad set of values related to a particular aspect of life (Kalberg, 2004), in this case, climate change. Consider worldview as people’s system of values that organise and integrate what they have learned about the world and themselves (e.g. through experience) in a symbolic representation system related to their views about climate change (Aerts; Van Belle; Van der Veken, 2012).
- **Another construct:** Is another construct part of the definition of perception? 0=No; 1=Yes.
- **Perception description:** how the perception concept appears in the results, discussion or conclusion, regardless of whether perception is defined.

1 = **Observation:** Perception is described as the OBSERVATION by an individual of changes in specific environmental aspects considered as EXTREME EVENTS OR ANOMALIE of climatic variability (e.g. individual reports changes—increases or decreases—in factors such as temperature, precipitation, drought and/or biophysical indicators, e.g., a person reports changes in plant flowering). Consider as extreme events or anomalies the following terms that may be accompanied by the words such as “extreme” or “anomalies”: storm, landslide, tornado, fire, drought, heatwave, cold wave, frost, forest fire, lightning, cyclone, hurricane, gale or flood.

2 = **Traditional Knowledge**: Perception is described through an individual's TRADITIONAL KNOWLEDGE of the weather or climate variability using a range of indicators or not that enable people to predict weather and climate. Traditional knowledge of climate change can include knowing what climate change is, its causes, and its effects when understood from forms of traditional knowledge. Consider as traditional knowledge when referred to this term in the article.

3 = **Scientific Knowledge:** Perception is described through an individual's SCIENTIFIC KNOWLEDGE of the weather or climate variability using meteorological information, information from scientists or computer models to predict weather and climate. This scientific knowledge of climate change can include knowing what it is, its causes, and its effects when obtained from these forms of scientific knowledge. Consider as scientific knowledge when it is referred like this in the article.

4 = **Information:** Perception is described through an individual's KNOWLEDGE of the weather or climate variability using information coming from people outside the community, such as from radio, television, newspapers, magazines, books or the internet. This knowledge of climate change can include knowing what climate change is, its causes, and its effects when obtained from these sources of information.

5 = **Concern:** Perception is described as the individual's degree of concern about the effects of climate change on livelihood activities. According to Kumar, Pandey & Anand, 2019, "livelihood is ability to obtain the basic necessities in life such as food, water, shelter, and clothing and all other necessities which required for human survival" (Kumar, Pandey & Anand, 2019, p.47).

6= **There is no description.**

7= **Another specific description, but not specified above**.

- **Definition description in results:** text or n⁄a (not applicable, i.e. when there is no description in the results). Copy exactly the sentence that contains the description of perception.
- **Observation of physical phenomenon:** Was the perception of a physical phenomenon reported in the article? 0=No; 1=Yes. Consider observing a physical phenomenon, the observation of changes or not in temperature, precipitation, wind, the emergence of extreme weather events or anomalies (e.g. drought, hurricane, fire, landslide, flood, tornado, among others), or changes in the seasons.
- **Observation of biological phenomenon:** Was the perception of a biological phenomenon reported in the article? 0=No; 1=Yes. Consider the observation of a biological phenomenon the observation of changes or not in plants (e.g., the flowering of a particular plant, vegetation cover, and invasive species), animals (e.g., the appearance of a specific animal, disappearance of insects, emergence of pests, invasive species) and other living beings.
- **Observation of human phenomenon:** Was the perception of a human phenomenon reported in the article? 0=No; 1=Yes. Consider the observation of any human phenomenon the observation of changes in subsistence practices (e.g., change in the period of performance of the activity or its non-performance); changes in human health (e.g., the emergence of diseases, basic sanitation); changes in the availability of food, human migration, or loss/gain of traditional knowledge.
- **Observation of another phenomenon:** Was the perception of another phenomenon reported in the article? 0=No; 1=Yes.

E. THEMATIC AREAS

- **Adaptation:** Does the article have adaptation as a theme? 0=No; 1=Yes. Consider adaptation as addressing measures, initiatives, coping strategies adopted or in the process of being planned to reduce or prevent the current and/or expected adverse effects of climate change, regardless of who is implementing them (affected population, government, NGOs, etc.). Consider an adaptation when referred to as adaptation or adaptive capacity or adaptive measure in the article.
- **Awareness:** Does the article have awareness as a theme? 0=No; 1=Yes. Consider awareness as the state or ability to perceive, feel, or be conscious of events, objects, or sensory patterns. Consider awareness when it is referred to as awareness in the article.
- **Traditional knowledge with indicators:** Does the article have traditional knowledge with indicators as a theme? 0=No; 1=Yes. Consider the articles with traditional knowledge when addressing physical (e.g., clouds, star position, atmospheric aspects) and biological indicators (e.g., animal behaviour, plant flowering, insect presence).
- **Traditional knowledge without indicators:** Does the article have traditional knowledge without indicators as a theme? 0=No; 1=Yes. Consider the pieces without traditional knowledge without addressing physical or biological indicators.
- **Scientific knowledge:** Does the article have scientific knowledge as a theme? 0=No; 1=Yes. Consider scientific knowledge when the paper presents information from meteorological data, on physical data, or any other data type gathered by scientists and researchers, or when it is referred to as scientific knowledge in the article.
- **Comparison between observations of individuals with scientific data:** Does the article compare individual observations with scientific data as a theme? 0=No; 1=Yes. For example, it compares meteorological or instrumental data with personal views about climate variability and/or climate anomalies.
- **Observed changes in livelihood activities:** Does the article have observed changes in the livelihood activities as a theme? 0=No; 1=Yes. Consider observed changes in livelihood activities the description of changes and their impacts on any aspect of people’s lives, such as on agricultural productivity, cash income, among others substance activities. According to Kumar, Pandey & Anand, 2019, "livelihood is ability to obtain the basic necessities in life such as food, water, shelter, and clothing and all other necessities which required for human survival" (Kumar, Pandey & Anand, 2019, p.47).
- **Observed changes in the environment:** Does the article describe the observed changes in the environment as a theme? 0=No; 1=Yes. Consider observed changes in the environment as the description of environmental changes (e.g., increased drought) and the environmental impacts resulting from these changes.
- **Determining factors**: Does the article investigate the determinants or drivers that may affect individual perceptions as a theme? 0=No; 1=Yes. Consider as determining factors the age, sex, education, monetary income, access to information, among others.
- **Mitigation:** Does the article have mitigation as a theme? 0=No; 1=Yes. Consider mitigation of the technological changes and/or other types of substitutions that reduce greenhouse gas emissions or that act to reduce these gases and aerosols. Consider as mitigation regardless of who is implementing them (affected population, government, NGOs, etc.).
- **Resilience:** Does the article have resilience as a theme? 0=No; 1=Yes. Consider as resilience the capacity of social, economic, and environmental systems to respond or cope in the face of a hazardous event, a trend or any disturbance. Consider resilience when individuals or groups respond to hazards or reorganise in ways that maintain their essential function, identity, and structure, while also maintaining the capacity for adaptation, learning, and transformation.
- **Risk perception:** Does the article have risk perception as a theme? 0=No; 1=Yes. Consider people’s subjective judgment about the characteristics and severity as risk perception.
- **Another thematic area:** Does the article have another thematic area as a theme? 0=No; 1=Yes.
- **What another thematic area:** describe another thematic area**.**

F. METHODS

- **Sampling unit:** sampling unit of collected data:

0 = **Not applicable**.

1 = **Individual level:** The sampling unit is the individual, i.e., subject.

2 = **Household level**: when the sampling unit is the household. Households refer to a group of individuals who live together, share the same food resources and aspects of consumption, and allocate a common set of resources (including work) to guarantee their material reproduction.

3 = **Individual and household level:** The article deals with two sampling units, the individual and the household.

4 = **Community or village:** when the sampling unit is a community of any type, i.e. referring to the residents of a community or a village. A community or village consists of a population cluster of a few houses in an environment that is not urban and economic activities at least partially rely on natural resources, including agriculture and animal husbandry. The community or village may be home to indigenous societies or smallholders. In this study, communities and villages do not refer to towns.

5 = **Organisations (e.g., NGOs, associations):** when the sampling unit is an organisation, such as associations, non-governmental and governmental organisations.

6 = **Another sampling unit not specified above.**

- **Data:** source of data used in the article for the analysis.

0 = **Not applicable.**

1 = **Primary data:** the researcher(s) collected in the field.

2 = **Secondary data:** not collected in the field by the researcher, coming from research institutions, government, other articles, among places other than the field.

3 = **Primary and secondary data: we will consider primary** and secondary data when the article deals with the two data types.

4 = **Another data source not specified above.**

- **Methods**: methods used in the article.

0 = **Not applicable.**

1 = **Qualitative methods:** e.g., informal interview, participatory approaches, semi-structured interview when you say it was qualitative, focus group, participant observation.

2 = **Quantitative methods:** e.g., structured interview, semi-structured interview, systematic observation.

3 = **Mixed methods**: include at least one qualitative and one quantitative method.

4 = **Another method type not specified above.**

G. THEORETICAL FOUNDATION

- **Theory of Planned Behavior:** Does the article adopt the Theory of Planned Behaviour as a framework of analysis or discussion? 0=No; 1=Yes.
- **Theory of Reasoned Action:** Does the article adopt the Theory of Reasoned Action as a framework of analysis or discussion? 0=No; 1=Yes.
- **Value-Believe-Norm (VBN):** Does the article adopt the Value-Belief-Norm framework for analysis or discussion? 0=No; 1=Yes.
- **Cognitive biases:** Does the article adopt any type of cognitive bias as a framework of analysis or discussion? 0=No; 1=Yes.
- **Theory of social norms:** Does the article adopt the Theory of Social Norms or the concept of social norms as a framework of analysis or discussion? 0=No; 1=Yes.
- **Cognitive dissonance theory:** Does the article adopt the Cognitive Dissonance Theory as a framework of analysis or discussion? 0=No; 1=Yes.
- **Another theory.**
- **What Theory:** describe which theory.

**REFERENCES**

Aerts D, Van Belle H, Van der Veken J: *World Views and the Problem of Synthesis: The Yellow Book of “Einstein Meets Magritte”*. Springer Science & Business Media; 2012.

Akerlof K et al. Do people “personally experience” global warming, and if so, how, and does it matter? Global environmental change. 2013:23(1):81-91.

Albarracin D, Shavitt S. Attitudes and attitude change. Annual review of psychology. 2018:69:299-327.

Andrade J: **Consciousness**. In: *Cognitive Psychology*. Edited by Braisby N, Gellatly A. Oxford University Press; 2012:577-605.

Ardoin N, et al: **Influencing Conservation Action: What Research Says about Environmental Literacy.** Behavior, and Conservation Results; 2013.

Arlt D, Hoppe I, Wolling J. Climate change and media usage: Effects on problem awareness and behavioural intentions. International Communication Gazette, 2011:73(1-2):45-63.

Chaiken S, Eagly AH. The Psychology of Attitudes. Cengage Learning; 1993:1-21.

Graham P, Graham E, Edgar H: **Perception**. In: *Cognitive* *psychology*. Edited by Braisby N, Gellatly A. Oxford University Press; 2012:65-99.

Hamilton-Webb A. et al. The relationship between risk experience and risk response: a study of farmers and climate change. Journal of Risk Research. 2017: 20(11):1379-1393.

IPCC. Annex II Glossary. WGIIAR5. Edited by Agard John et al. 2014:1757-1776.

Iturriza M, et al. Shifting to climate change aware cities to facilitate the city resilience implementation. Cities. 2020: 101: 102688.

Kalberg S. The past and present influence of world views: Max Weber on a neglected sociological concept. Journal of Classical Sociology. 2004:4(2):139-163.

Kumar H, Pandey BW, Anand S. Analyzing the impacts of forest ecosystem services on livelihood security and sustainability: a case study of Jim Corbett National Park in Uttarakhand. International Journal of Geoheritage and Parks. 2019:7(2):45-55.

Paton D, et al. Direct and vicarious experience of volcanic hazards: implications for risk perception and adjustment adoption. The Australian Journal of Emergency Management. 2000:15(4): 58-63.

Poortinga W, et al. Climate change perceptions and their individual-level determinants: A cross-European analysis. Global Environmental Change. 2019:55:25-35.

Raymond CM, et al. Integrating local and scientific knowledge for environmental management. Journal of environmental management. 2010:91(8):1766-1777.

Sánchez MJ, Lafuente R. Definición y medición de la conciencia ambiental. Revista internacional de sociología. 2010:68(3):731-755.

Sternberg RJ, Sternberg K, Mio J: *Cognitive psychology*. Cengage Learning Press; 2012.

Triantafyllidou E, Zabaniotou A. From Theory to Praxis: ‘Go Sustainable Living’ Survey for Exploring Individuals Consciousness Level of Decision-Making and Action-Taking in Daily Life Towards a Green Citizenship. Circular economy and sustainability. 2021:1-27.
